# Chemically Crosslinked Methylcellulose Substrates for Cell Sheet Engineering

**DOI:** 10.3390/gels7030141

**Published:** 2021-09-14

**Authors:** Lorenzo Bonetti, Luigi De Nardo, Silvia Farè

**Affiliations:** 1 Department of Chemistry, Materials and Chemical Engineering “G. Natta”, Politecnico di Milano, Via Luigi Mancinelli 7, 20131 Milan, Italy; luigi.denardo@polimi.it (L.D.N.); silvia.fare@polimi.it (S.F.); 2 National Interuniversity Consortium of Materials Science and Technology (INSTM), Via Giuseppe Giusti 9, 50121 Florence, Italy

**Keywords:** methylcellulose, citric acid, crosslinking, thermoresponsive hydrogels, cell sheet engineering (CSE)

## Abstract

Methylcellulose (MC) hydrogels have been successfully proposed in the field of cell sheet engineering (CSE), allowing cell detachment from their surface by lowering the temperature below their transition temperature (T_t_). Among the main limitations of pristine MC hydrogels, low physical stability and mechanical performances limit the breadth of their potential applications. In this study, a crosslinking strategy based on citric acid (CA) was used to prepare thermoresponsive MC hydrogels, with different degrees of crosslinking, to exploit their possible use as substrates in CSE. The investigated amounts of CA did not cause any cytotoxic effect while improving the mechanical performance of the hydrogels (+11-fold increase in E, compared to control MC). The possibility to obtain cell sheets (CSs) was then demonstrated using murine fibroblast cell line (L929 cells). Cells adhered on crosslinked MC hydrogels’ surface in standard culture conditions and then were harvested at selected time points as single CSs. CS detachment was achieved simply by lowering the external temperature below the T_t_ of MC. The detached CSs displayed adhesive and proliferative activity when transferred to new plastic culture surfaces, indicating a high potential for regenerative purposes.

## 1. Introduction

Cell sheet engineering (CSE) has emerged as a newsworthy approach in the tissue engineering and regenerative medicine fields [1,2,3]. CSE is a scaffold-free approach that allows overcoming some of the shortcomings associated with conventional scaffold-based approaches (e.g., biodegradation rate, host inflammatory response, stress shielding) [4,5]. Moreover, for regenerative purposes, CSE represents an advantage over the use of cell suspensions, in which a significant cell loss occurs after injection due to the low cell retention rates at the site of interest. Cell sheets (CSs) obtained via the CSE approach, retaining their deposited extracellular matrix (ECM), rich of adhesive proteins, have been successfully delivered to the host tissues and wound sites with minimal cell loss [6,7].

In this regard, thermoresponsive methylcellulose (MC) hydrogels are ideal substrates for the obtainment of CSs [8,9,10,11]. Specifically, the transition temperature (T_t_) can be conveniently set so that at T < T_t_ MC is in a sol, hydrophilic state governed by hydrogen bonds between water molecules and the hydrophilic groups (-OH) in the MC macromolecules. Conversely, at T ≥ T_t_ MC is in a gel, hydrophobic state, governed by hydrophobic interactions among the hydrophobic groups (-CH_3_) of MC [12]. Cells cultured on MC hydrogels surface can be harvested as intact CSs simply by lowering the external temperature, avoiding the use of proteolytic enzymes, and thus preserving intact cell–cell junctions and the deposited ECM [12,13].

However, MC hydrogels display some major limitations [12]. According to previous research [9,14,15,16], an 8% MC hydrogel prepared in 50 mM Na_2_SO_4_ displays a T_t_~37 °C, ideal for CSE. Nevertheless, it shows limited physical stability in the water environment (25% residual weight 7 days after immersion in dH_2_O) and low mechanical performance (G’~10^1^–10^2^ Pa at 37 °C) [14]. In fact, when long-term cell cultures are expected, MC hydrogels’ fast dissolution could impair their application. Furthermore, their low mechanical properties could fail to recapitulate the adequate environment required to guide the fate and functions (e.g., adhesion, spreading, differentiation) of the seeded cells [17] and the development of adequate CSs.

Chemical crosslinking, inducing the formation of covalent bonds among MC chains, can be regarded as an effective strategy to overcome the abovementioned limitations [12]. On this topic, different crosslinking methods have been proposed for MC crosslinking (e.g., photocrosslinking [18,19,20], γ or electron beam irradiation [21,22], small molecule crosslinkers such as glutaraldehyde [21,23], divinyl sulfone [24], or citric acid [14,25]). However, the crosslinking process must be optimized in order not to alter the thermoresponsive behavior of the crosslinked MC gels. In fact, chemical crosslinking has been reported to cause shifts in the T_t_ or even impair the responsive behavior of thermoresponsive hydrogels [12]. We previously reported the crosslinking of MC hydrogels by means of citric acid (CA) as an effective way to obtain hydrogels with superior physical and mechanical performances (i.e., reduced degradation in water environment and increased rheological parameters) by preserving thermoresponsiveness [14]. However, the possibility to obtain CSs from CA-crosslinked MC hydrogels has never been explored thus far.

In this work, we investigated the suitability of CA-crosslinked MC hydrogels in the CSE approach. Three different crosslinking degrees were analyzed and compared to MC control hydrogels. The obtained hydrogels were explored from a physical, mechanical, and biological point of view. First, swelling tests in water were carried out to assess the effectiveness of CA crosslinking in modulating the water uptake of MC gels. Additionally, a Flory–Rehner model was applied to the swelling data to calculate the main theoretical physical parameters describing the microstructure of the crosslinked gels. Then, tensile tests were carried out to assess Young’s modulus (E) ranges of the MC gels. In vitro, indirect cytotoxicity was then carried out to exclude possible cytotoxic effects induced using CA as a crosslinker. Subsequently, L929 murine fibroblasts were seeded on the crosslinked MC samples, then confluent cell sheets were harvested from their surfaces simply by decreasing the external temperature from 37 to 4 °C. Lastly, the adhesion and proliferation of the cells constituting the CSs were qualitatively evaluated in vitro to assess the regenerative potential of the obtained CSs.

## 2. Results and Discussion

### 2.1. MC Hydrogels

MC represents an optimal choice to produce thermoresponsive surfaces for CSE because of its low cost, simplicity of preparation, and easy tunability of its thermoresponsive behavior [8]. In particular, the accurate selection of MC concentration and the addition of salting-out ions are the key aspects to be considered to decrease the T_t_ toward a range of interest for CSE applications (i.e., T_t_ about 37 °C); indeed, a 2% MC solution shows a typical T_t_~60 °C [12]. In this regard, 8% MC and 50 mM Na_2_SO_4_ were selected based on previous studies [9,16] to obtain a T_t_ = 37 °C.

Even if the transition temperature can easily be tuned by the addition of salting-out ions [16,26], the main limitations of MC hydrogels consist of their reduced water stability (i.e., fast dissolution) and low mechanical properties in physiological conditions. We previously reported a possible approach to MC crosslinking, based on citric acid (CA), for the obtainment of chemically crosslinked MC hydrogels with preserved thermoresponsive character [14]. In the present work, three optimized crosslinked MC hydrogel formulations (Table 1) were investigated for CSE purposes, comparing them to pristine MC hydrogels (i.e., used as control). In what follows, the differently crosslinked MC hydrogels will be referred to as MC-L, MC-M, MC-H, meaning low, medium, and high crosslinking degree, respectively. Table 1 also reports the content of citric acid (i.e., the crosslinking agent), the theoretical carboxyl content, and the weight fraction of CA taking part in ester bonds (w_ester_, [14]) for each formulation here investigated. As it is possible to observe, the w_ester_ of MC-L samples is lower (0.6%) than that of MC-M (51.3%) and MC-H (77.6%) samples, suggesting a low crosslinking degree of MC-L samples [14].

### 2.2. Swelling Tests

**Figure 1:**
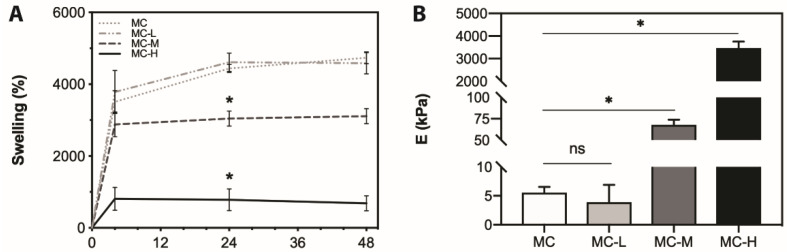
(**A**) Swelling behavior of the crosslinked MC specimens. The swelling equilibrium was identified at t = 24 h. * = *p* < 0.05, compared to MC control; (**B**) Young’s modulus (E) of CA-crosslinked MC hydrogels. * = *p* < 0.05. Pristine MC hydrogel was used as control.

Figure 1A displays the swelling behavior of the crosslinked MC hydrogels within 48 h of incubation in distilled water at 37 °C. All the hydrogels showed a weight increase within the first 24 h, due to water absorption. After 24 h, a swelling plateau was reached for all the formulations, indicating that the swelling equilibrium was reached.

As it is possible to notice, the increase in the crosslinking degree of the samples causes a significant reduction (*p* < 0.05) in their swelling values. In particular, the swelling values at the swelling equilibrium were shown to be different (*p* < 0.05) when comparing the crosslinked MC hydrogels with MC control, with average swelling values ranging from ~800% for MC-H to 3000% for MC-M. Conversely, MC-L did not show significant differences (*p* > 0.05) in terms of swelling at the equilibrium, compared to MC control. This suggests that low crosslinking slightly influenced the swelling behavior of the specimens [14]. In fact, an increase in the crosslinking degree causes an increase in crosslinking points, preventing the crosslinked MC network expansion in the water environment.

According to the Flory–Rehner model [14,27,28,29], it is possible to calculate the key parameters (Supplementary Material S1) defining the microstructure of crosslinked hydrogel networks, i.e., the average molecular weight between crosslinking points ($\bar{M_{C}}$), the crosslinking density ($\rho_{C}$), and the mesh size (ξ), reported in Table 2. Focusing on the crosslinking density (Table 2), it is possible to observe a ~12-fold increase in terms of $\rho_{C}$ from MC control (0.20 ± 0.01 × 10^4^ mol cm^−3^) to MC-H samples (2.42 ± 0.87 × 10^4^ mol cm^−3^). This indicates that the crosslinking degree of MC samples can be effectively tuned via a CA-based crosslinking procedure [14]. Similar results have also been obtained on methacrylated MC hydrogels crosslinked with reduction–oxidation (redox) initiators, such as ammonium persulfate (APS)–ascorbic acid (AA) initiation system [29] or APS-N,N,N’,N’-tetramethylethylenediamine (TEMED) initiation system [30]. Interestingly, comparing the mesh size of these systems, ξ values in the 40–80 nm range were observed for MC crosslinked with APS–AA redox system [29], while ξ was in the 30–70 nm range in APS–TEMED redox polymerization reactions [30]. The abovementioned ranges are in accordance with the ξ values obtained in this work, ranging from 10 to 60 nm. Slight differences in the ξ values between this work and the abovementioned studies can be attributed to the different crosslinking agents used, the different M_w_ of MC (88 kDa vs. 14 kDa), and the different MC concentrations used.

### 2.3. Tensile Tests

The mechanical properties of MC hydrogels were tested on hydrated (i.e., swelling equilibrium) samples to mimic the cell culture conditions. Figure 1B reports Young’s moduli (E) calculated for each sample; it is possible to observe that the MC hydrogels stiffness was effectively modulated by increasing the crosslinking degree. As expected, highly crosslinked samples (MC-H) display the highest elastic modulus (E = 3.47 ± 0.29 MPa), while MC and MC-L samples display the lowest values (E = 5.53 ± 1.00 kPa and 3.90 ± 2.98 kPa, respectively). In particular, MC-L samples do not show any significant difference, compared to MC control, indicating poor effectiveness of CA crosslinking when a low CA concentration is used (Table 2). MC-M samples display E values midway between MC and MC-H (E = 67.83 ± 6.03 kPa), with a ~11-fold increase, compared to control (MC).

These outcomes are in accordance with previous rheological data on the same crosslinked MC samples, revealing an increase in the viscoelastic parameters (G’ and η*) as a function of the crosslinking degree [14]. In this regard, the storage modulus (G’) of MC-H samples, at T = 37 °C, was three orders of magnitude higher than that of MC. Similarly, Figure 1B displays a three order-of-magnitude increase in E values when comparing the highest crosslinking degree (MC-H) with MC control. The E values of the crosslinked MC hydrogels were obtained around their T_t_, thus representing the values that these samples would display in cell culture environment (T = 37 °C, samples at the swelling equilibrium).

The tunability of the stiffness of a substrate for CSE can be relevant to design surfaces able to influence the overall response of the cultured cells. On this topic, it is widely accepted that mechanical (e.g., stiffness, viscosity) cues are essential parameters in surface design to modulate biological processes and even control cell fate (e.g., attachment, spreading, differentiation), similar to biochemical signals [31,32]. In particular, Young’s modulus of biological tissues ranges from 100 Pa (e.g., neural tissue) to 10 GPa (e.g., bone tissue) [33]. CA-crosslinked MC hydrogels would represent self-standing substrates capable to provide adequate mechanical cues to the seeded cells. Conversely, poly(N-isopropylacrlamide) (PNIPAAm), the reference material in the CSE field, needs to be grafted on a substrate with defined mechanical properties (e.g., E), to provide adequate cues to the seeded cells [34,35].

The first step toward the obtainment of substrates for CSE capable to provide the predetermined mechanical properties while preserving their thermoresponsive behavior consists of verifying that CA-crosslinked MC substrates allow on-demand (i.e., temperature-triggered) harvesting of CSs.

### 2.4. In Vitro Biological Tests

#### 2.4.1. Indirect Cytotoxicity

**Figure 2:**
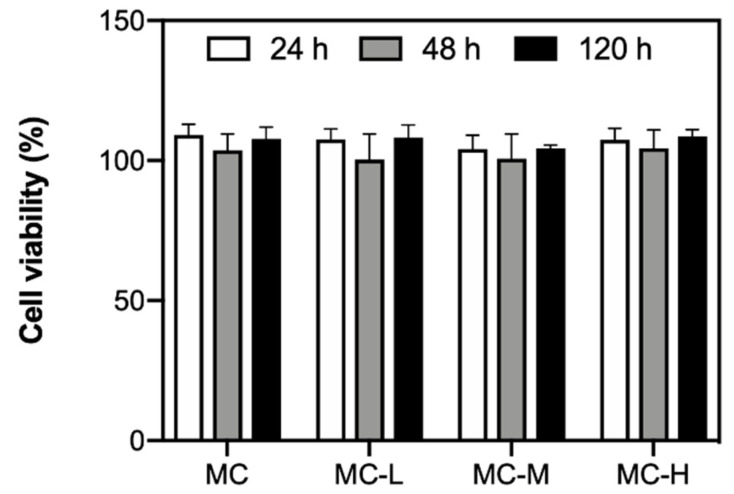
In vitro cytotoxicity test: viability of L929 cells kept in contact with 24, 48, and 120 h eluates of crosslinked MC hydrogel (pristine MC used as control).

Cell viability around 100 % was observed for each crosslinking condition (Figure 2), comparable to pristine MC, with no statistical differences (*p* > 0.05) among the samples.

These data suggest that the CA amounts used in our study (up to 5% w_CA_/w_MC_ for MC-H samples) did not cause any cytotoxic effect on L929 cells. Our data are in accordance with previous studies in the literature exploiting CA as crosslinking agent [36,37,38]. In this regard, a concentration of CA up to 20% (w_CA_/w_polymer_) has been demonstrated to be non-toxic yet effective to achieve crosslinking of different polymers (e.g., polyvinyl alcohol/starch [36], collagen [37]).

It should be noted that the efficiency of crosslinking is never 100% for all the crosslinked MC samples. In fact, according to our previous results on the same hydrogel formulations, the amount of -COOH of CA involved in ester bonds (i.e., w_ester_, Table 1) ranged from ~1% to ~80%, increasing the crosslinking degree (i.e., MC-L to MC-H samples) [14]. This means that residual (i.e., non-reacted) citric acid is present after the crosslinking procedure. However, in this study, the samples were not washed after the crosslinking procedure, since a washing step would have removed the Na_2_SO_4_ added during the preparation of MC hydrogels. Such removal would have caused a shift in the T_t_ (due to the removal of salting-out anions [12]) of the MC hydrogels toward higher values, thus preventing their use for CSE purposes. Regardless, the amount of non-reacted CA in the investigated samples in this work was found to be non-cytotoxic (Figure 2), as demonstrated by cell viability values >70 % (i.e., the non-cytotoxicity threshold according to the standard ISO 10993–5:2009, “Biological evaluation of medical devices—Part 5: Tests for in vitro cytotoxicity”). Overall, these results suggest the possible use of crosslinked MC hydrogels as substrates for CSE.

#### 2.4.2. Cell Sheets Harvesting

**Figure 3:**
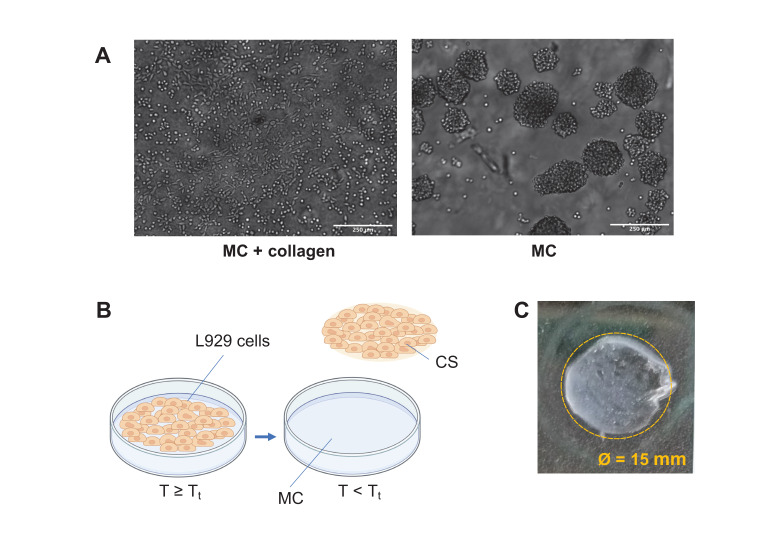
(**A**) Effect of rat tail type I collagen coating on cell adhesiveness of MC 24 h after seeding. Pristine MC, without collagen absorption (right) does not support the adhesion of L929 cells, which form aggregates on the hydrogel surface. Conversely, L929 cells seeded on collagen-coated MC adhere and spread on the MC surface (left). Scale bar = 250 μm; (**B**) scheme of CS harvesting: at 37 °C (i.e., T ≥ T_t_), L929 cells adhere and proliferate on the MC surface, due to its moderately hydrophobic nature. At T < 37 °C, MC changes to hydrophilic, allowing the spontaneous detachment of an intact CS; (**C**) representative image of an intact L929 CS detached from the surface of a thermoresponsive MC hydrogel.

The scarce protein adsorption and limited cell adhesiveness of MC hydrogels make it necessary to implement strategies to open their use as platforms for CSE [8]: different approaches have been proposed to specifically address cell adhesion [8,9,12,16,39,40,41]. For instance, the immobilization of laminin−1, as a bio-adhesive ligand, has been employed on in situ gelling MC hydrogels for neural tissue engineering [39]. Another possible approach to improve cell adhesion consists of blending MC with other polymers (e.g., agarose [40], collagen [41]). In the field of CSE, the surface of each MC hydrogel is usually modified with collagen absorbed prior to cell seeding [8,9,16]. In this work, rat tail type I collagen was adsorbed on the surface of MC hydrogels as a preliminary stage to increase cell adhesion. Figure 3A (right) displays the poorly adhesivity of MC surface without collagen, which leads to the formation of cell aggregates. Conversely, rat tail type I collagen absorption resulted in L929 cell adhesion and spreading on the surface of the MC hydrogels Figure 3A (left). These observations are in accordance with previous studies in the literature on the role of type I collagen absorption on MC surface in effectively promoting cell adhesion [8,42].

L929 cells on collagen-treated MC hydrogels were cultured for 24 and 120 h. Under culture conditions (i.e., T = 37 °C), thermoresponsive MC surfaces are relatively hydrophobic, allowing cells to attach and proliferate. CS detachment was achieved by lowering the external temperature to 4 °C (T < T_t_) for 20 min. In these conditions, MC structure changes to hydrophilic, forming a hydration layer between the cells and the MC surface. Thus, cells spontaneously detach as an intact CS (Figure 3B), without the need for any treatment (e.g., trypsin-based treatment) [9]. CSs with a diameter of 15 mm (Figure 3C) were harvested from MC, MC-L, and MC-M hydrogels. Conversely, the harvesting was not feasible from the surface of MC hydrogels with the highest crosslinking degree (MC-H). This supports our previous results, which displayed how excessive crosslinking (i.e., MC-H) leads to the loss of the hydrogel thermoresponsiveness [14]. This phenomenon has been demonstrated to be caused by the formation of a dense crosslinking network, which limits MC chains mobility, thus constraining the formation of weak interactions (i.e., H-bonds among water and MC chains, and CH_3_-CH_3_ bonds among MC chains) responsible for the thermoresponsive behavior of MC hydrogels [14].

Due to the impossibility to detach CSs from MC-H substrates, for the subsequent characterization only MC, MC-L, and MC-M formulations were considered.

#### 2.4.3. Cell Sheets Characterization

**Figure 4:**
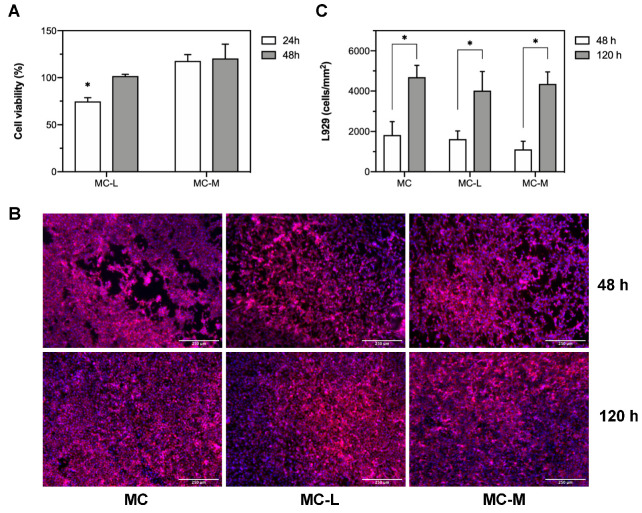
(**A**) L929 metabolic activity on MC hydrogels after culture for 24 and 48 h. MC was used as control. * = *p* < 0.05 compared to MC control; (**B**) fluorescence micrographs of L929 CSs detached from MC, MC-L, and MC-H hydrogels, 48 and 120 h after seeding. Scale bar = 250 μm; (**C**) cell density (cells/mm^2^) calculated from image analysis, for MC, MC-L, and MC-H hydrogels, 48 and 120 h after seeding. * = *p* < 0.05.

L929 metabolic activity was investigated 24 and 48 h after cell seeding on the MC hydrogels under study (Figure 4A). The resazurin assay revealed cell viability around 100 % for both MC-L and MC-M samples when compared to control (MC), indicating evident proliferation and viability of cells in contact with the MC hydrogels. A significantly lower (*p* < 0.05) cell viability was detected for MC-L samples 24 h after seeding, probably due to a delayed cell proliferation on this substrate, compared to MC control. However, at t = 48 h, cell viability was found comparable to the one of MC control.

Fluorescence micrographs of the CSs after 48 and 120 h of culture (Figure 4B) qualitatively revealed the presence of widespread red areas, indicating the actin filaments in the cytoskeleton, also contributing to cell–cell junction in the obtained CSs [9]. The produced CSs appeared compact, indicating a good cell cohesiveness reached in the culture time length. Moreover, it was possible to qualitatively observe an increase in cell density between 48 and 120 h cultured CSs.

The cell density was quantitatively assessed by means of image analysis, which confirmed the aforesaid qualitative observations. Specifically, a significant (*p* < 0.05) increase in cell density was observed for all the MC substrates, between 48 and 120 h of cell culture (Figure 4C). The theoretical cell density for 48 h cultured CSs was calculated by considering the cell seeding density (1.5 × 10^5^ cells/well) and L929 cells division time (~14 h, [43]). The theoretical cell density after 48 h is ~9000 cells/mm^2^ (Supplementary Material S3), higher than the measured one ~2000 cells/mm^2^ (Figure 4C). This difference can be explained by the fact that L929 cells were already close to confluency due to the high seeding density (Supplementary Material S4) Additionally, it is possible to observe (Figure 4C) that, despite the extended culture time, only a twofold increase in the cell density values was achieved for 120 h cultured CSs compared to 48 h cultured CSs. This small increase in cell density values for all MC samples further confirmed the fact that the L929 cells have reached confluency between 48 and 120 h [13]. For each cell culture time (48 or 120 h), no significant differences in terms of cell density were observed among the CSs obtained from differently crosslinked samples (MC, MC-L, and MC-M).

**Figure 5:**
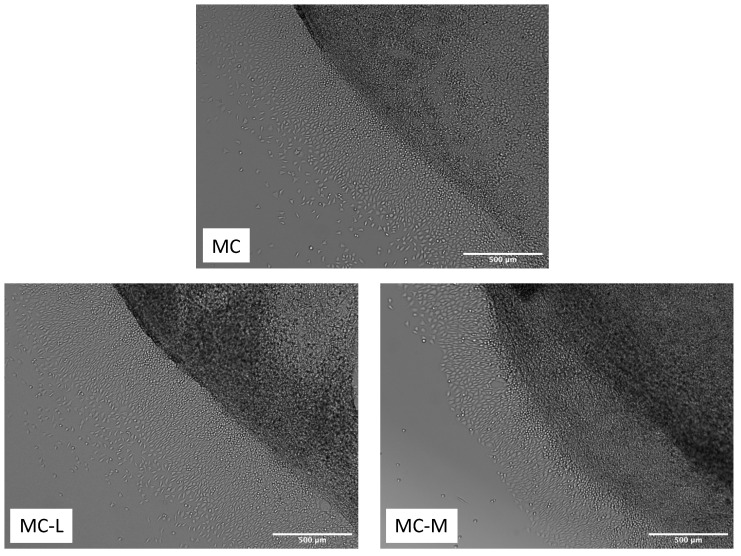
CSs cultured for 72 h on a new substrate (12-MW). Cell migration and proliferation from each CS (upper right side) to the well bottom (lower left side) can be observed in the shading degree of each image. Scale bar = 500 μm.

For each crosslinked MC substrate, two CSs were transferred to a new 12-well plate (12-MW) to qualitatively assess the CSs adhesive and proliferation activities on a new substrate. The CSs started to adhere to the new substrates shortly after their transfer (~20 min). Adhesion was confirmed, as the CSs remained on the bottom of the wells after the addition of further DMEM (i.e., 250 μL/well, used to extend the CSs culture time up to 72 h). Interestingly, 72 h after their transfer, CSs qualitatively preserved their closely interconnected structure, indicating that cell–cell junctions have not been destroyed after CSs harvesting from the MC hydrogel surfaces. Moreover, optical microscopy observation 72 h after CSs transfer (Figure 5) revealed that the L929 cells started to migrate from the CS to the bottom of the 12-MW wells. No qualitative differences in cell adhesion and proliferation were observed among the CSs obtained from the crosslinked MC substrates and MC control.

Taken together, these observations disclose the potential of CSs obtained from crosslinked MC thermoresponsive hydrogels for tissue regeneration [9]. The obtained CSs, avoiding the use of scaffolding materials as cell carriers, could prevent materials-related complications (e.g., host inflammatory response). Moreover, by preserving intact cell–cell junctions and their deposited ECM, they could be implanted in the host tissues without the need for any mediators (e.g., fibrin glue or sutures) [44,45]. Complex tissues could also be regenerated by multiple transplants of CSs (even of different cell types) in the host tissues or by transplanting in vitro-layered CS [46].

The obtained results show that CSs detached from the surface of crosslinked MC hydrogels displayed no qualitative and quantitative differences, compared with control MC samples. It is well documented how the growth of L929 cells is insensitive to the stiffness of the culture surface, supporting that the capability to sense or respond to mechanical cues may be impaired in some cell types, e.g., immortalized or cancer cells [47,48,49]. On this topic, Mih et al. revealed the insensitiveness of L929 cells to substrates with stiffnesses in the 0.1–100 kPa range [49], comparable to the one investigated in the present study. Moreover, even substrates with higher E ranges (i.e., in the order of MPa) have been reported not to influence the spreading and the shape of L929 cells seeded on them [50]. Since the present study was intended as a bench test for crosslinked MC substrates to evaluate their responsive behavior in the field of CSE, the obtained outcomes should not be considered unfavorable. On the contrary, these outcomes identify CA-crosslinked MC hydrogels as noteworthy substrates for CSE applications. In fact, in addition to the possibility to harvest CSs, they offer unique advantages, compared to pristine MC hydrogels. First, their increased water stability would allow their use for prolonged cell culture times, at the state-of-the-art limited by the fast dissolution of pristine MC hydrogels in the water environment [9,14]. Moreover, the tuning of the crosslinking parameters allowed obtaining MC substrates with E values in the 5–75 kPa range (MC to MC-M) and with preserved thermoresponsive character. Such substrates can be regarded as promising self-standing platforms with tunable mechanical performances, useful, for instance, to guide the fate of selected cell types (e.g., mesenchymal stem cells) and obtain mature (i.e., differentiated) CSs [31]. Nevertheless, further tuning of the crosslinking process, exploring crosslinking conditions midway between MC-M and MC-H, could provide thermoresponsive substrates with superior performances, compared to MC-M hydrogels (representing, in this work, the upper limit in terms of preserved thermoresponsive character).

## 3. Conclusions

In this work, we demonstrated the feasibility of obtaining CSs from CA-crosslinked MC substrates. CSs were successfully detached from optimized crosslinked MC hydrogels, despite excessive crosslinking (i.e., MC-H) leading to the loss of the thermoresponsive behavior of MC, thus preventing CSs detachment. The CSs obtained from crosslinked MC hydrogels displayed no differences in terms of viability, adhesive, and proliferative activities, compared to the CSs obtained from MC control.

Overall, these results open the floodgates to the obtainment of stable (e.g., to aqueous or cell culture environment) MC hydrogels, suitable for prolonged cell culture times. Moreover, CA-crosslinked MC hydrogels could represent self-standing and mechanically tunable substrates for CSE, which lend themselves to guiding and regulating the fate of the seeded cells (e.g., stem cells).

## 4. Materials and Methods

Unless stated, all chemicals were purchased from Sigma-Aldrich (Milan, (MI), Italy) and used as received without further purification.

### 4.1. MC Hydrogels Preparation

Methylcellulose (MC) hydrogels were prepared according to a previously reported protocol [14]. Briefly, an 8% *w/v* MC (M0512) suspension was prepared by dispersing MC powder in a 0.05 M Na_2_SO_4_ deionized water solution, at 55 °C. Citric acid (CA) (Sigma-Aldrich, Milan, Italy), the crosslinking agent, was added in different concentrations (1, 3, or 5% w_CA_/w_MC_) to the solution. The obtained suspensions were dispensed (T = 4 °C, t = 24 h) into Petri dishes (Euroclone, Pero, Italy), then oven-dried (T = 50 °C, t = 24 h) to obtain MC films. Lastly, MC crosslinking was achieved by thermal treatment, varying the crosslinking time and temperature according to Table 3. Non-crosslinked MC samples were used as controls.

### 4.2. Swelling Tests

The swelling properties of MC hydrogels were investigated by placing dry MC samples (*n* = 3 for each formulation) into cell strainers (mesh size = 40 μm, Corning, New York, NY, USA) and immersing them in distilled water (dH_2_O) at 37 °C. At different time points (i.e., 4, 24, 48 h), the strainers were withdrawn from the water, gently blotted on tissue paper to remove excess water, then weighted. The swelling ratio (SW) was calculated according to the following equation (Equation (1)):

(1) $$SW\;\left(\%\right)=\frac{w_{t}-w_{o}}{w_{0}}\times \;100$$

where *w_t_* and *w*_0_ represent the weight at time t (i.e., wet weight), and the weight at time 0 (i.e., dry weight) of an MC sample, respectively. The swelling equilibrium was considered when no significant differences in terms of SW were detected among two subsequent time points.

### 4.3. Mechanical Tensile Tests

MC samples (*n* = 3 for each formulation) at the swelling equilibrium (i.e., t = 24 h for all samples, T = 37 °C, in dH_2_O [14]) were tested by Dynamic Mechanical Analyzer (DMA Q800, TA Instruments, New Castle, DE, USA), equipped with tension clamps. A preload of 0.001 N was applied, followed by a strain-controlled ramp at a 0.3 min^−1^ rate. Young’s modulus (E) was calculated from the stress–strain curves as the slope in the 0–5% strain range (R^2^ > 0.95).

### 4.4. In Vitro Biological Tests

MC dry films were UV sterilized (30 min/side) prior to in vitro testing. Samples were then immersed in sterile distilled water, at 37 °C, for 24 h to reach the swelling equilibrium.

#### 4.4.1. Indirect Cytotoxicity

In vitro indirect cytotoxicity tests were performed to assess the possible release of toxic compounds from the crosslinked MC hydrogels. Complete Dulbecco’s modified Eagle medium (DMEM) with the addition of 1 mM sodium pyruvate, 10 mM HEPES buffer, 100 U mL^−1^ penicillin, 0.1 mg mL^−1^ streptomycin, 2 mM glutamine, and 10% (*v/v*) fetal bovine serum was used as an extraction medium. Circular MC hydrogel samples (Φ = 15 mm) were obtained by a manual punch. Each specimen (*n* = 3 for each crosslinking condition and each time point) was immersed in the extraction medium (3 cm^2^ 1 mL^−1^ extraction ratio, according to the ISO 10993-12 standard for samples with thickness in the 0.5 < t < 1 mm range) and then incubated at 37 °C for (24, 48, and 120) h. At each time point, eluates were harvested; aged (24, 48, and 120 h) DMEM culture medium, without MC, was used as control (CTRL).

L929 murine fibroblasts (*n* 85011425, ECACC, Public Health England, Salisbury, UK) were seeded in a 96-well plate (cell density = 1 × 10^4^ cells/well) and cultured with 100 μL of complete DMEM for 24 h. Then, DMEM was replaced with eluates or control culture media (100 μL), and cells were incubated for a further 24 h in standard culture conditions. Cell metabolic activity was assessed by resazurin assay [16]. Fluorescence was measured with a Synergy H1 spectrophotometer (BioTek, Santa Clara, CA, USA; λ_ex_ = 540 nm, λ_em_ = 595 nm) for cells cultured in the eluates (RFU_sample_) and in the aged culture medium (RFU_CRTL_), after background fluorescence subtraction of the resazurin solution incubated without cells (RFU_resazurin_). For each well, cell viability was calculated according to the following equation (Equation (2)):


(2) $$Viability\;\left(\%\right)=\left[\frac{RFU_{sample}-RFU_{resazurin}}{RFU_{CTRL}-RFU_{resazurin}}\right]\times \;100$$

#### 4.4.2. Cell Culture

For each crosslinking condition, circular hydrogel specimens (Φ = 20 mm) were obtained by a manual punch. The hydrogel specimens (*n* = 3 for each crosslinking condition) were positioned on the bottom of the tissue-culture multiwell plate (12 wells, 12-MW, Euroclone, Pero, (MI), Italy), and kept in position by means of polydimethylsiloxane (PDMS, Elastosil RT 601, Wacker, Munich, Germany) rings (Φ_EXT_ = 22 mm, Φ_INT_ = 15 mm, Supplementary Material S2). Prior to cell seeding, 200 μL of type I collagen extracted from rat tail (4 mg mL^−1^) were adsorbed on the surface of each hydrogel specimen to enhance cell adhesion [9]. L929 cells were seeded (1.5 × 10^5^ cells/well in 100 μL DMEM [9]) on each specimen and cultured in standard culture conditions for 48 or 120 h.

##### CSs Harvesting

After 24 or 120 h of culture, the plates were removed from the incubator and placed at 4 °C for 20 min. This procedure allowed the produced CSs to spontaneously detach from the MC hydrogel surface (Figure 3B,C). CSs were collected by adding PBS at T = 4 °C into each well and sucking them up with a 50 mL pipette. The obtained CSs were then moved onto electrostatic glass slides or into a new 12-MW well for immunofluorescence staining and adhesion/proliferation tests, respectively.

##### Resazurin Assay

L929 metabolic activity was investigated by performing tests on cells cultured on MC specimen (*n* = 3 for each crosslinking condition), 24 and 48 h after seeding, by means of the resazurin assay, as previously described. Previous in vitro cytocompatibility tests on non-crosslinked hydrogel formulation (i.e., MC) revealed good proliferation and viability of cells in contact with MC hydrogels [9]. Thus, cells cultured on MC hydrogels were used as control. For each well, cell viability was calculated according to the following equation (Equation (3)):

(3) $$Viability\;\left(\%\right)=\left[\frac{RFU_{MC-X}-RFU_{resazurin}}{RFU_{MC}-RFU_{resazurin}}\right]\times \;100$$

where MC-X = MC-L, MC-M, or MC-H.

##### Immunofluorescence Staining

The detached CSs (*n* = 1 for each crosslinking condition) were transferred to electrostatic slides, rinsed with PBS solution, then fixed with a 4% (*v/v*) paraformaldehyde solution in PBS for 20 min at room temperature. After rinsing with PBS solution, the CSs were permeabilized with 0.2 % (*v/v*) Triton X-100 in PBS for 20 min at room temperature. Each CS was then stained (60 min at room temperature) with 100 μL of a 0.2 % (*v/v*) Triton X-100 solution in PBS containing phalloidin (P1951, 4 μg/mL) to stain actin filaments, and Hoechst (Hoechst 33342, ThermoFisher Scientific, Waltham, MA, USA, 1 μg/mL) to stain the nuclei. After rinsing with PBS, 2–3 drops of Fluoromount (F4680) were applied on each CS, and a coverslip was gently applied. The specimens were observed with a fluorescent microscope (Zeiss Axioplan, Oberkochen, Germany). Cell density (cells mm^−2^) was calculated by image analysis (ImageJ, v. 1.53, NIH, National Institutes of Health, Bethesda, MD, USA), counting the average number of nuclei in the acquired images (*n* = 5 for each CS) and dividing it by the area of the images (0.148 mm^2^, Supplementary Material S3).

##### Adhesion and Proliferation on a New Substrate

CSs detached from MC hydrogels (*n* = 2 for each crosslinking condition) were transferred to a new 12-MW to qualitatively assess CSs adhesion and proliferation on a new substrate. A total of 50 μL of DMEM was added to each CS to avoid dehydration. After 20 min, an additional 250 μL of DMEM was added to each well, and CSs were cultured in standard culture conditions. After 72 h of culture, CSs were observed by optical microscope (Zeiss Axioplan) to evaluate the potential L929 cells migration outside of each CS.

### 4.5. Statistical Data Analysis

All tests were carried out in triplicate (*n* = 3). Data are reported as mean ± standard deviation (SD). Statistical analysis (GraphPad Prism v 8.0, San Diego, CA, USA) was performed using single *t*-tests or one-way ANOVA tests, with significance level *p* = 0.05.

## Figures and Tables

**Table 1:** MC hydrogel formulations: CA content, carboxyl content, and weight fraction of CA taking part in ester bonds.

Sample	Crosslinking Degree	[CA] (% w_CA_/w_MC_)	-COOH (mmol/100 g)	w_ester_ * (%)
MC	-	0	0	0
MC-L	Low	1	15.6	0.6
MC-M	Medium	3	46.9	51.3
MC-H	High	5	78.1	77.6

* = Data taken from [14].

**Table 2:** Theoretical physical parameters defining the microstructure of the crosslinked MC hydrogels. $\bar{M_{C}}$ = average molecular weight between crosslinking points, $\rho_{C}$ = crosslinking density, and ξ = mesh size. *, #, and † = significantly different (*p* < 0.05), compared to MC control.

Sample	$\bar{M_{C}}\;(g\;{\mathrm{mol}}^{-1})$	$\rho_{C}\;(\mathrm{mol}\;{\mathrm{cm}}^{-3})\;\times \;10^{-4}$	ξ (nm)
MC	1.36 × 10^4^ ± 5.33 × 10^2^	0.20 ± 0.01	59.98 ± 1.64
MC-L	1.45 × 10^4^ ± 1.25 × 10^3^	0.19 ± 0.02	62.53 ± 3.77
MC-M	7.72 × 10^3^ ± 7.70 × 10^2^ *	0.36 ± 0.04 #	40.21 ± 2.84 †
MC-H	1.28 × 10^3^ ± 5.77 × 10^2^ *	2.42 ± 0.87 #	11.29 ± 3.51 †

**Table 3:** MC samples prepared and characterized in this work. MC = non-crosslinked sample. MC-L = low crosslinking; MC-M = medium crosslinking; MC-H = high crosslinking.

Sample	[CA] (% w_CA_/w_MC_)	Temperature (°C)	Time (min)
MC	-	-	-
MC-L	1	165	1
MC-M	3	177.5	8
MC-H	5	190	15

## Supplementary Materials

The following are available online at https://www.mdpi.com/article/10.3390/gels7030141/s1, Supplementary Material S1: Flory–Rehner model, Supplementary Material S2: Plate scheme for CSs production, Supplementary Material S3: Cell density, Supplementary Material S4: Confluency.
