# Supplementary material for: Chemically Crosslinked Methylcellulose Substrates for Cell Sheet Engineering
Source: Gels. 2021 Sep 14;7(3):141. doi: 10.3390/gels7030141 (PMC8482237; doi:10.3390/gels7030141)
Supplement: Supplementary file 1 [file gels-07-00141-s001.zip › gels-1356209-supplementary.pdf]

gels

Supplementary Materials

Chemically crosslinked methylcellulose substrates for cell sheet engineering

Lorenzo Bonetti [1], Luigi De Nardo [1,2] and Silvia Farè [1,2]

[1] Department of Chemistry, Materials and Chemical Engineering "G. Natta", Politecnico di Milano, Via Luigi Mancinelli 7, 20131 Milan, Italy; luigi.denardo@polimi.it (L.D.N.); silvia.fare@polimi.it (S.F)
[2] National Interuniversity Consortium of Materials Science and Technology (INSTM), Via Giuseppe Giusti 9, 50121, Florence, Italy;
* Correspondence: lorenzo.bonetti@polimi.it; Tel.: +39 02 2399 4741

Supplementary Material S1: Flory-Rehner model

The theoretical physical parameters describing the microstructure of MC gels, namely the average molecular weight between crosslinking points ($\overline{M_C}$), the crosslinking density (ρ_C), and the mesh size (ξ), were calculated according to a simplified version of the Flory-Rehner model [1–4].

In particular, the average molecular weight between crosslinking points ($\overline{M_C}$) was calculated using the following equations (Eq. S1 - S3) [1,3–5]:

$$Q_v^{5/3} \cong \frac{\bar{v}\overline{M_C}}{v_1}\left(\frac{1}{2}-\chi\right) \quad \text{(Eq. S1)}$$

$$Q_v = 1 + \left(\frac{\rho_p}{\rho_s}(Q_w - 1)\right) \quad \text{(Eq. S2)}$$

$$Q_w = \frac{w_S}{w_D} \quad \text{(Eq. S3)}$$

where w_S and w_D are the weights of the MC samples in swollen or dry conditions, respectively. Q_w and Q_v represent the equilibrium weight swelling ratio and the volumetric swelling ratio, respectively. The other terms (i.e., constants) of the equations, are reported in **Error! Reference source not found.**.

The crosslinking density (ρ_C) was calculated according to the following equation (Eq. S4) [1,3,4]:

$$\rho_C = \frac{1}{\bar{v}\overline{M_C}} \quad \text{(Eq. S4)}$$

The constant values of Eq. S4 are reported in **Error! Reference source not found.**.

The mesh size (ξ) of the hydrogel at the swelling equilibrium was calculated using the following equation (Eq. S5) [1,3,4]:

$$\xi = 0.217\sqrt{\overline{M_C}}Q_v^{1/3} \quad \text{(Eq. S5)}$$

Table S1. Constant terms used in Eq. S1 – S4.

Term	Symbol	Value	Ref
Density of dry polymer	ρ_p	0.276 g cm^{-3}	[1,6]
Density of the solvent	ρ_s	1 g cm^{-3}	
Specific volume of dry polymer	$\bar{v} = \frac{1}{\rho_p}$	3.623 cm^3 g^{-1}	
Molar volume of the solvent	v_l	18 mol cm^{-3}	
Flory polymer-solvent interaction parameter	χ	0.473	[1,4]

gels

Supplementary Material S2: Plate scheme for CSs production

Figure S1 reports the scheme of the 12-well plate (12-MW) for CSs production. MC hydrogel specimens (Φ = 20 mm) are obtained by manual punch. The specimens are kept on the bottom of the well by using PDMS rings (Φ_{EXT} = 22 mm, Φ_{INT} = 15 mm, h = 2 mm).

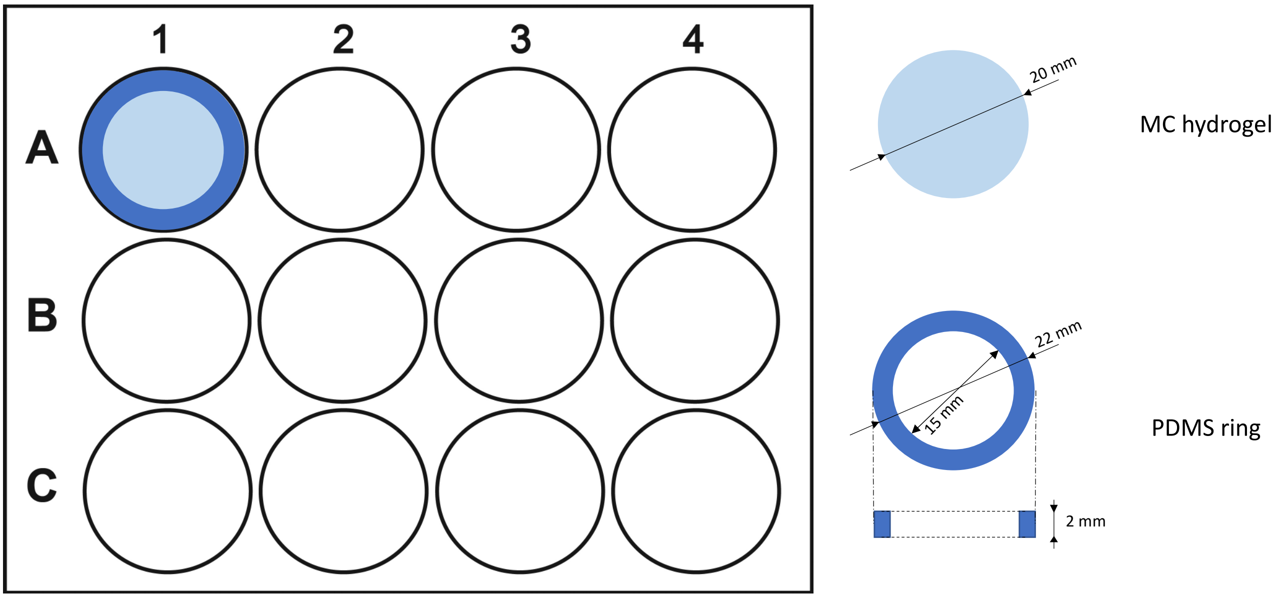

Figure S1 – 12-MW plate scheme for CSs production.

Supplementary Material S3: Cell density

2A. Theoretical cell density

Given

- cell seeding density: 1.5×10^5 cells/well
- L929 doubling time: 14 h [7]
- cell culture time: 48 h
- cell divisions in cell culture time: 3.42
- culture surface: 176.7 mm^2 (i.e., hydrogel surface, $\Phi = 15$ mm, **Figure S1)**

The theoretical cell density after 48 h of culture was calculated as follows:

$$\text{Cell density (cells/mm}^2)_{48h} = \underbrace{(1.5 \times 10^5) \times 2^{3.42}}_{\text{exponential growth equation [8]}} \text{ cells} / 176.7 \text{ mm}^2 = 9086 \text{ cell/mm}^2$$

2B. Calculated cell density

Figure S2 A displays a representative cell count performed on two fluorescence images of 48- and 120 h cultured CSs. Cell density (cells/mm^2) (**Figure S2 B**) was calculated by image analysis (ImageJ, v. 1.53, NIH), counting the average number of nuclei in the acquired images ($n = 5$ for each CS) and dividing it by the area of the images (0.148 mm^2).

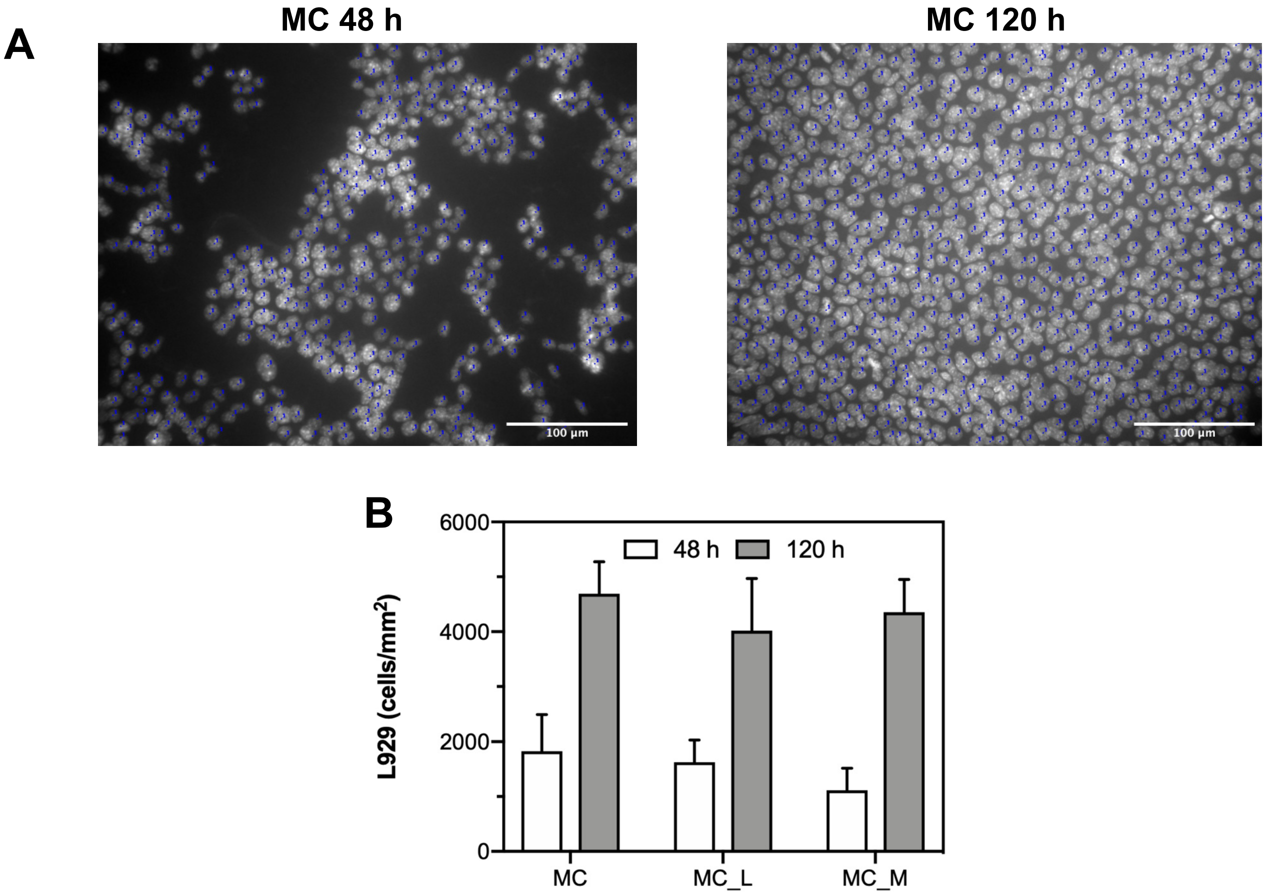

Figure S2 – A) Representative nuclei counting from fluorescence images of 48 and 120 h-cultured CSs. Scale bar = 100 µm. B) L929 cell density calculated from image analysis.

Supplementary Material S4: Confluency

Figure S3 display representative images of L929 cells cultured on MC hydrogel substrates (pristine MC and crosslinked MC) 48 h after seeding. It is possible to observe that cells are close to confluency, due to the high cell density selected for the obtainment of CSs. Some cells are unfocused because of the non-planarity of the MC substrates.

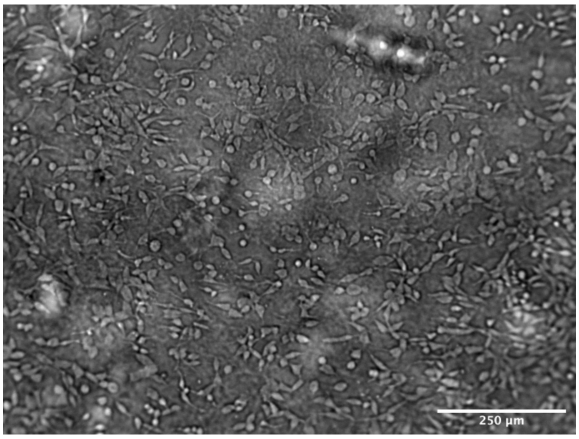

MC

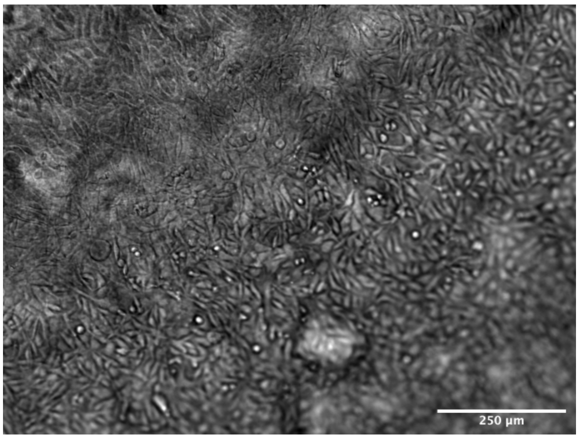

MC-L

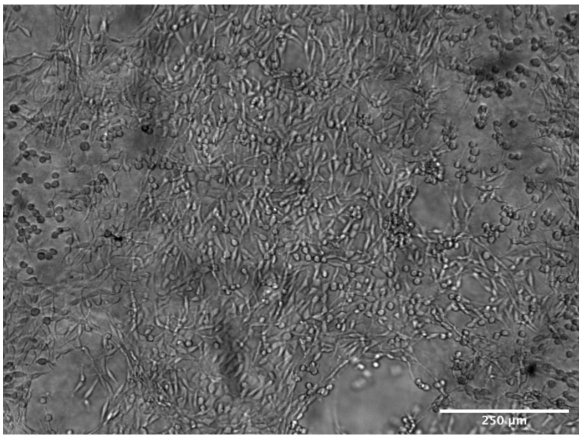

MC-M

Figure S3 – L929 cultured on MC substrates 48 h after seeding. Scale bar = 500 μm.

References

1. Bonetti, L.; De Nardo, L.; Variola, F.; Fare', S. Evaluation of the subtle trade-off between physical stability and thermo-responsiveness in crosslinked methylcellulose hydrogels. *Soft Matter* **2020**, *16*, 5577–5587.
2. Flory, P.J.; Rehner, J. Statistical Mechanics of Cross-Linked Polymer Networks II. Swelling. *J. Chem. Phys.* **1943**, *11*, 521–526.
3. Baier Leach, J.; Bivens, K.A.; Patrick Jr., C.W.; Schmidt, C.E. Photocrosslinked hyaluronic acid hydrogels: Natural, biodegradable tissue engineering scaffolds. *Biotechnol. Bioeng.* **2003**, *82*, 578–589.
4. Gold, G.T.; Varma, D.M.; Taub, P.J.; Nicoll, S.B. Development of crosslinked methylcellulose hydrogels for soft tissue augmentation using an ammonium persulfate-ascorbic acid redox system. *Carbohydr. Polym.* **2015**, *134*, 497–507.
5. Stalling, S.S.; Akintoye, S.O.; Nicoll, S.B. Development of photocrosslinked methylcellulose hydrogels for soft tissue reconstruction. *Acta Biomater.* **2009**, *5*, 1911–1918.
6. Rowe, R.C.; Sheskey, P.J.; Quinn, M.E. *Handbook of Pharmaceutical Excipients*; 6th Editio.; Pharmaceutical Press: London, 2009; ISBN 9780853697923.
7. Bucur, M.; Constantin, C.; Neagu, M.; Zurac, S.; Dinca, O.; Vladan, C.; Cioplea, M.; Popp, C.; Nichita, L.; Ionescu, E. Alveolar blood clots and platelet-rich fibrin induce in vitro fibroblast proliferation and migration. *Exp. Ther. Med.* **2018**.
8. Sherley, J.L.; Stadler, P.B.; Stadler, J.S. A quantitative method for the analysis of mammalian cell proliferation in culture in terms of dividing and non-dividing cells. *Cell Prolif.* **1995**, *28*, 137–144.
